# RTN1A mediates diabetes-induced AKI-to-CKD transition

**DOI:** 10.1172/jci.insight.185826

**Published:** 2024-12-20

**Authors:** Lulin Min, Ya Chen, Yixin Chen, Fang Zhong, Zhaohui Ni, Leyi Gu, Kyung Lee, John Cijiang He

**Affiliations:** 1 Department of Nephrology, Renji Hospital, School of Medicine, Shanghai Jiao Tong University, Shanghai, China.; 2 Department of Medicine/Nephrology, Icahn School of Medicine at Mount Sinai, New York, New York, USA.; 3 Renal Section, James J. Peters Veterans Affair Medical Center, Bronx, New York, USA.

**Keywords:** Nephrology, Chronic kidney disease, Diabetes, Mitochondria

## Abstract

Diabetic patients have increased susceptibility to acute kidney injury (AKI), and AKI could progress to chronic tubulointerstitial injury and fibrosis, referred to as AKI-to–chronic kidney disease (AKI-to-CKD) transition. However, whether diabetes directly promotes AKI-to-CKD transition is not known. We previously showed that reticulon-1A (*RTN1A*), a gene highly upregulated in injured renal tubular epithelial cells (RTECs), promotes AKI-to-CKD transition in nondiabetic settings. Therefore, we also examined whether reducing RTN1A expression could attenuate diabetes-induced AKI-to-CKD transition. Diabetes was induced by a high-fat diet and streptozotocin injections, and unilateral ischemic reperfusion injury was created as an AKI model in control, diabetic, and RTEC-specific *Rtn1a*-knockdown diabetic mice. AKI induced greater renal function decline, tubulointerstitial injury, and fibrosis in diabetic mice than in nondiabetic mice. Reduction of RTN1A markedly reduced the CKD development following AKI in diabetic mice, which was associated with reduced ER stress and mitochondrial dysfunction in RTECs. These findings indicate that diabetes markedly accelerates AKI-to-CKD transition and that RTN1A is a crucial mediator of diabetes-induced AKI-to-CKD transition. The development of RTN1A inhibitors could potentially attenuate AKI-to-CKD transition in diabetic patients.

## Abstract 2

Underlying diabetes increases susceptibility to chronic kidney disease development, which is in part driven by RTN1A protein-mediated kidney tubular cell injury.

## Introduction

Recent United States Renal Data System (USRDS) data indicate that approximately 40% of diabetic patients have chronic kidney disease (CKD) and that 60% of end-stage kidney disease (ESKD) patients are diabetic, indicating diabetic kidney disease (DKD) remains the leading cause of ESKD in the United States (USRDS annual data report 2023). Despite the renoprotection conferred by the newly approved medications, such as sodium-glucose transporter 2 inhibitors, mineralocorticoid receptor antagonists, and glucagon-like peptide-1 receptor agonists (1), many patients continue to progress toward ESKD due to substantial residual risk for DKD progression.

There is a growing recognition that DKD includes a broader spectrum of disease than classically defined diabetic nephropathy, based on findings of histopathologic glomerular lesions on kidney biopsies, and encompasses both glomerular and non-glomerular kidney damage induced by diabetes. Indeed, a substantial proportion of DKD patients who present minimal albuminuria (i.e., nonalbuminuric DKD) manifest relatively normal glomerular structures with mild glomerular basement membrane thickening and mesangial expansion (2–4), but predominantly display tubulointerstitial injury and fibrosis. However, the pathogenesis of nonalbuminuric DKD is incompletely understood. Notably, a higher incidence of AKI is associated with diabetes in humans (5–7), and experimental models have demonstrated a causal link between diabetes and increased susceptibility to ischemic AKI (8). Reciprocally, as maladaptive repair following AKI promotes irreversible CKD, referred to as AKI-to-CKD transition and marked by ongoing tubulointerstitial injury and fibrosis (9), ischemic AKI in the diabetic milieu may be a crucial instigator of transition into nonalbuminuric DKD. However, this has not been formally demonstrated using experimental models; thus, potential mechanisms are not yet defined.

We previously identified reticulon-1A (RTN1A) as an instigator of tubular injury and CKD progression (10). RTN1A localizes primarily on the endoplasmic reticulum (ER) membrane as an ER-shaping protein, and its expression is markedly increased in renal tubular epithelial cells (RTECs) in AKI and CKD. Notably, its expression inversely correlates with renal function in diabetic patients (10–12), and 3 intronic SNPs of *RTN1* are associated with ESKD in diabetic patients (13). We previously demonstrated that RTEC-specific RTN1A knockdown mitigated AKI-to-CKD transition in mouse models of folic acid– and aristolochic acid–induced kidney damage (11, 14). However, whether RTN1A is involved in diabetes-mediated AKI-to-CKD transition has not been explored to our knowledge. Elevated expression of RTN1A in kidney injury settings promotes marked tubular damage by inducing sustained ER stress and mitochondrial dysfunction, leading to RTEC apoptosis (15). As ER stress and mitochondrial dysfunction are both involved in the development and progression of DKD (16–21), we reasoned that increased RTN1A expression in diabetic kidneys may underlie AKI-to-CKD transition following ischemic injury, potentially leading to nonalbuminuric DKD. Therefore, in this study, we assessed the contribution of underlying diabetes in promoting AKI-to-CKD transition and RTN1A’s role in this process.

## Results

### Diabetes predisposes mice to aggravated tubular damage after uIRI, which is abrogated by RTN1A inhibition.

**Figure 1:**
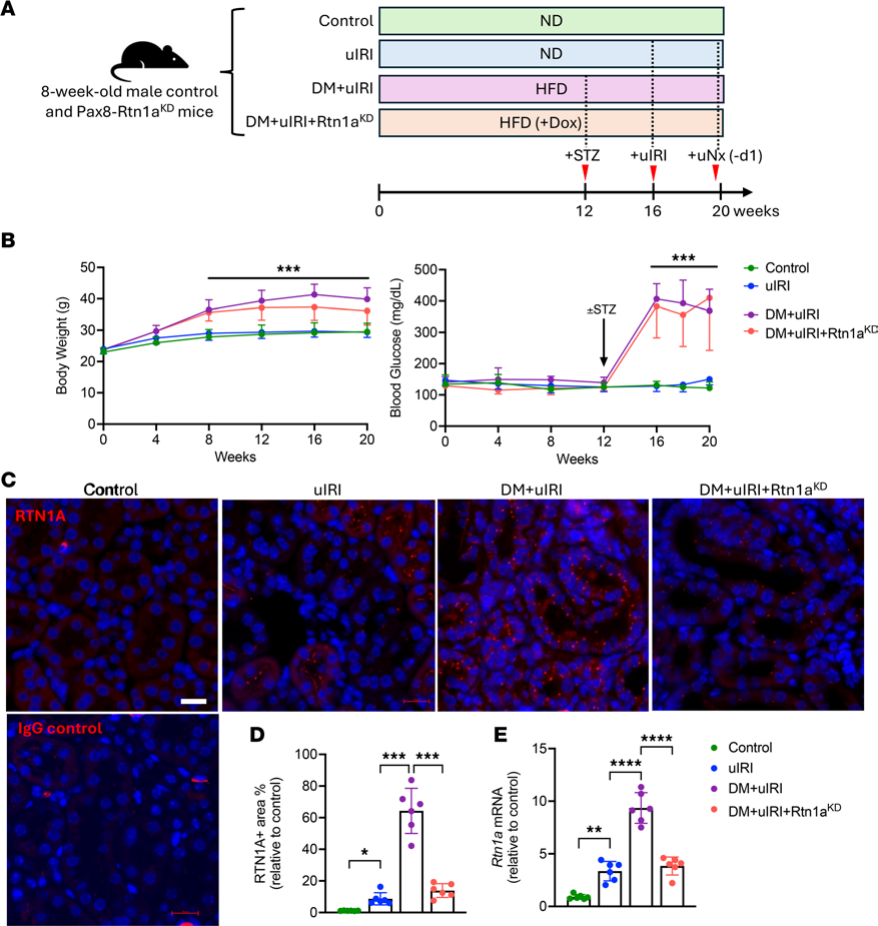
RTN1A expression is increased in RTECs by ischemic and diabetic tubular damage. (**A**) Schematics of the experimental outline. Six 8-week-old male C57BL/6J mice were randomized into 4 experimental groups consisting of control mice, mice with unilateral ischemic reperfusion injury (uIRI group), diabetic mice with uIRI (DM+uIRI), and diabetic mice with uIRI with *Rtn1a* knockdown (DM+uIRI+*Rtn1a*^KD^). Diabetes was induced by high-fat diet (HFD) supplementation and low-dose streptozotocin (STZ) injections. Nondiabetic mice were given a normal diet (ND) with vehicle injections. All mice were euthanized after 20 weeks of each diet, and contralateral kidneys were removed 1 day (uNx, –d1) before endpoint analysis to assess kidney function in mice subjected to uIRI. (**B**) Body weight and blood glucose levels in the 4 groups are shown. Body weight change was statistically significant in diabetic mice starting at 8 weeks of HFD supplementation, and blood glucose levels were significantly elevated after STZ injection in diabetic mice compared with nondiabetic mice. (**C**) Representative RTN1A immunofluorescence images of mouse kidney sections. Negative control with IgG control is shown on the bottom. Nuclei are counterstained with DAPI. Scale bar: 20 μm. (**D**) Quantification of RTN1A^+^ area is shown as fold change relative to the Control group (*n* = 6 mice per group, 30 fields evaluated per mouse). (**E**) Real-time PCR analysis of total *Rtn1a* mRNA expression with primers that detect both mouse and human transcripts (*n* = 6 mice per group). *P < 0.05; **P < 0.01; ***P < 0.001; ****P < 0.0001 between indicated groups by 2-way ANOVA with Dunnett’s post hoc test (**B**) or 1-way ANOVA with Tukey’s post hoc test (**D** and **E**).

We previously developed a doxycycline-inducible RTEC-specific *Rtn1a*-knockdown model, resulting in approximately 80% inhibition of *Rtn1a* expression (*Rtn1a*^KD^) (10, 11, 14). To examine whether diabetes would predispose mice toward accelerated CKD transition after ischemic AKI, diabetes was first induced in *Rtn1a*^KD^ and control littermate mice (lacking one or more transgenes), and mice were subjected to unilateral ischemic reperfusion injury (uIRI). As *Rtn1a*^KD^ and control littermates are in the mixed C57BL/6J background that is more resistant to DKD development, diabetes was induced by the administration of a high-fat diet (HFD) for 12 weeks, followed by low-dose streptozotocin (STZ) injections, as shown in Figure 1A. This experimental model is useful in capturing the aspects of both dyslipidemia and insulin deficiency seen in both type 1 and type 2 diabetic individuals, as approximately 60% of type 1 diabetic patients develop obesity, and type 2 diabetic patients often have both obesity and insulin deficiency (22, 23). Nondiabetic groups of mice were administered normal diet and vehicle injections. All mice on HFD gradually gained significant body weight by 8 weeks of HFD, but showed prominent hyperglycemia only after receiving STZ injections (Figure 1B). RTEC-specific knockdown of *Rtn1a* in diabetic mice did not affect body weight gain or blood glucose levels (Figure 1B). uIRI was induced in control and diabetic mice by clamping the right renal pedicle for 30 minutes at 37°C 4 weeks after STZ or vehicle injection. All mice were euthanized at 20 weeks after initiation of HFD feeding (after 4 weeks of uIRI) (Figure 1A). Unilateral nephrectomy (uNx) of the contralateral (left) kidneys was performed in uIRI mice 1 day before euthanasia to determine the renal function of the ischemic kidney. Thus, the experimental groups consisted of (a) nondiabetic control mice without AKI (Control), (b) nondiabetic mice with uIRI (uIRI), (c) diabetic mice with uIRI (DM+uIRI), and (d) diabetic *Rtn1a*^KD^ mice with uIRI (DM+uIRI+*Rtn1a*^KD^).

Since RTN1A expression increases in RTECs in human DKD and after AKI and is associated with the severity of tubular injury (10, 11), we first examined the expression of RTN1A in mouse kidneys. As expected, immunofluorescent staining indicated that RTN1A expression was hardly detectable in control mouse kidneys, but significantly increased in RTECs by uIRI and even more so by diabetes and uIRI (Figure 1, C and D). RTN1A knockdown effectively reduced its expression in diabetic mice with uIRI, but without a complete abrogation. The change in RTN1A expression was further validated by quantitative PCR (qPCR) of mouse kidney cortices (Figure 1E).

**Figure 2:**
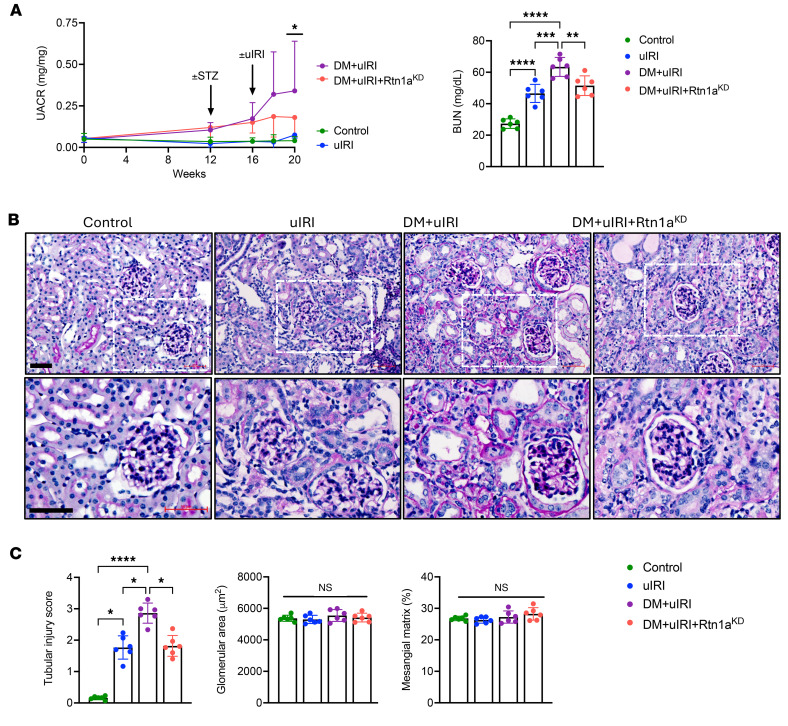
Diabetes predisposes mice to worsened kidney function after uIRI, which is attenuated by RTN1A knockdown. (**A**) Kidney function assessment by urinary albumin-to-creatinine ratio (UACR) and blood urea nitrogen (BUN) levels. uIRI significantly accelerated albuminuria development in DM+uIRI mice, but not in DM+uIRI+*Rtn1a*^KD^ when examined at endpoint. BUN levels were significantly elevated in all mice with uIRI compared with controls, but only further heightened in DM+uIRI mice (*n* = 6 mice). (**B**) Representative images of PAS-stained kidneys. Dotted areas in the top panels are magnified in the bottom panels. Dashed lines in the top panels are magnified in the bottom panels. Scale bars: 50 μm. (**C**) Quantification of average tubular injury score, glomerular area, and percentage mesangial matrix area are shown (*n* = 6 mice, 30 fields evaluated for tubular injury score per mouse). *P < 0.05; **P < 0.01; ***P < 0.001; ****P < 0.0001 between indicated groups by 2-way ANOVA with Dunnett’s post hoc test (**A**, left), 1-way ANOVA with Tukey’s post hoc test (**A**, right), or Kruskall-Wallis test with Dunn’s post hoc test (**C**, left). Forty glomeruli were evaluated for glomerular area and mesangial fraction. NS by 1-way ANOVA with Tukey’s post hoc test (**C**, right).

We next examined the kidney function in the 4 groups of mice. At 20 weeks, urine albumin was modestly elevated in both groups of diabetic mice when compared with nondiabetic mice (Figure 2A), but significantly less urine albumin was detected in DM+uIRI+*Rtn1a*^KD^ mice compared with DM+uIRI mice. Significant elevation in blood urea nitrogen (BUN) levels was observed in all mice subjected to ischemic injury, but this was substantially augmented in DM+uIRI mice compared with uIRI mice (Figure 2A). Notably, DM+uIRI+*Rtn1a*^KD^ mice, despite having diabetes, displayed BUN levels comparable to nondiabetic uIRI mice, suggesting that the aggravation of tubular damage by diabetes after AKI is abrogated by the reduction in RTN1A. Indeed, the histopathologic analysis of mouse kidneys was consistent with these observations. Periodic acid–Schiff–stained (PAS-stained) kidneys showed sustained tubular injuries at 4 weeks after ischemic injury in uIRI mice, consistent with incomplete recovery after AKI (Figure 2, B and C). Notably, increased severity of tubulointerstitial damage was observed in DM+uIRI mice, with atrophic tubules and thickened tubular basement membranes, which was substantially reduced in DM+uIRI+*Rtn1a*^KD^ mice (Figure 2, B and C). However, at this early DKD stage (8 weeks after STZ injection), the diabetic mice did not yet show any overt glomerular lesions (Figure 2, B and C). Thus, it is likely that the aggravated tubular damage in DM+uIRI mice contributes to the further increase in albuminuria observed at 20 weeks in comparison with DM+uIRI+*Rtn1a*^KD^ mice (Figure 2A).

### Diabetes predisposes mice to increased fibrosis, inflammation, and tubular senescence after uIRI, which is abrogated by RTN1A inhibition.

**Figure 3:**
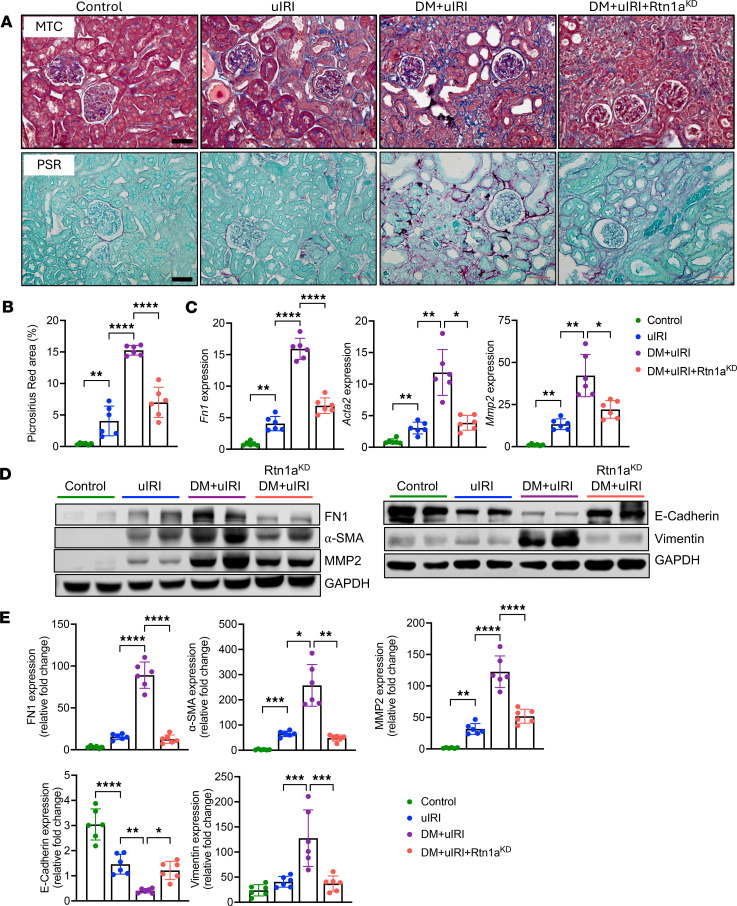
Diabetes predisposes mice to increased renal fibrosis after uIRI, which is attenuated by RTN1A knockdown. (**A**) Representative images of kidney sections stained with Masson’s trichrome (MTC) and picrosirius red (PSR) with fast green staining. Scale bar: 50 μm (original magnification, ×200). (**B**) Quantification of PSR-stained fibrotic area (%). Thirty fields were evaluated per mouse. (**C**) Real-time PCR analysis of fibrosis markers (*Fn1*, *Acta2*, and *Mmp2*) in mouse kidney cortices. (**D**) Representative Western blot analysis of fibrosis markers (FN1, α-SMA, and MMP2) and epithelial or mesenchymal markers (E-cadherin or vimentin, respectively) in mouse kidney cortices. (**E**) Densitometric analysis of proteins in 3D shown as a fold change relative to Control mice (*n* = 6 mice). *P < 0.05; **P < 0.01; ***P < 0.001; ****P < 0.0001 between indicated groups by 1-way ANOVA with Tukey’s post hoc test.

**Figure 4:**
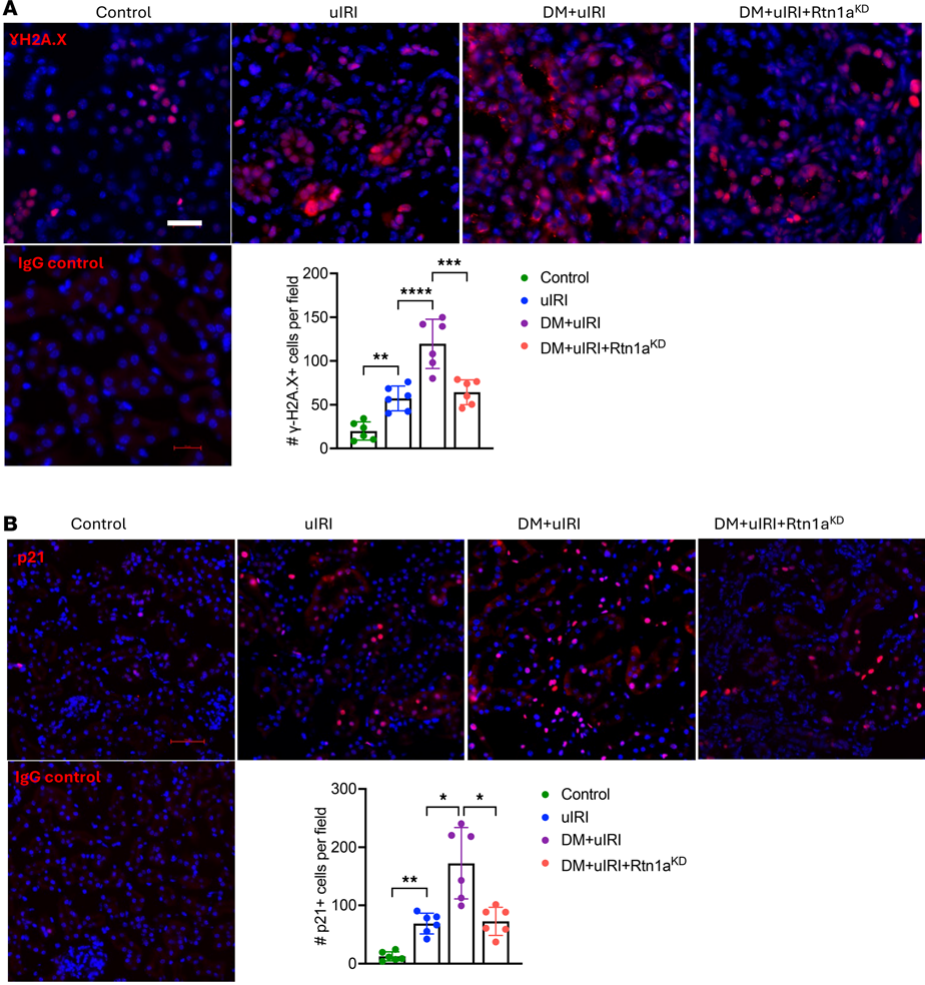
Diabetes exacerbates RTEC senescence in mice with uIRI, which is attenuated by RTN1A knockdown. (**A**) Representative images of γ-H2A.X immunofluorescence and quantification. Scale bar: 20 μm. (**B**) Representative images of p21 immunofluorescence and quantification. Scale bar: 50 μm. *n* = 6 mice, 30 fields analyzed per mouse. *P < 0.05; **P < 0.01; ***P < 0.001; ****P < 0.0001 between indicated groups by 1-way ANOVA with Tukey’s post hoc test.

Given the marked tubulointerstitial damage sustained after uIRI in diabetic mice, we next examined the effects of diabetes on the ensuing renal fibrosis development. Masson’s trichome and picrosirius red with fast green staining showed mild renal fibrosis in the nondiabetic uIRI mice, but much more pronounced fibrosis development in DM+uIRI mice (Figure 3, A and B). RTN1A knockdown markedly attenuated renal fibrosis in diabetic mice with uIRI, consistent with reduced tubular injury. We further validated the histologic findings with the expression of fibrosis markers, fibronectin (FN1), α-smooth muscle actin (α-SMA), and matrix metallopeptidase 2 (MMP2) by qPCR and Western blot analyses. Both analyses confirmed a significant increase in the expression of profibrotic markers in DM+uIRI mouse kidneys in comparison with uIRI, and that RTN1A knockdown counteracted these changes (Figure 3, C–E). We also observed an increased expression of vimentin and reduced expression of E-cadherin in kidney cortices of DM+uIRI mice, reflecting a partial de-differentiation of RTECs (Figure 3, D and E), which were also normalized by RTN1A knockdown. Another important aspect of the AKI-to-CKD transition is the increased senescence of tubular epithelial cells due to maladaptive repair response (7). Immunofluorescent staining of senescence markers, γ-H2A.X and p21, showed a marked increase in these markers in DM+uIRI mice (Figure 4, A and B), which was mitigated by RTN1A knockdown. Consistent with these findings, RTN1A knockdown also reduced the severity of renal inflammation in diabetic mice with uIRI, as shown by F4/80 immunostaining and qPCR of inflammatory cytokines (Supplemental Figure 1; supplemental material available online with this article; https://doi.org/10.1172/jci.insight.185826DS1). These results are consistent with the above findings that underlying diabetes promotes and accelerates AKI-to-CKD transition by aggravating tubular damage, fibrosis, and inflammation and that RTN1A is a major culprit in this process.

### ER stress and mitochondrial dysfunction in RTECs underlie the accelerated AKI-to-CKD transition in diabetic mice, which are reversed by RTN1A inhibition.

**Figure 5:**
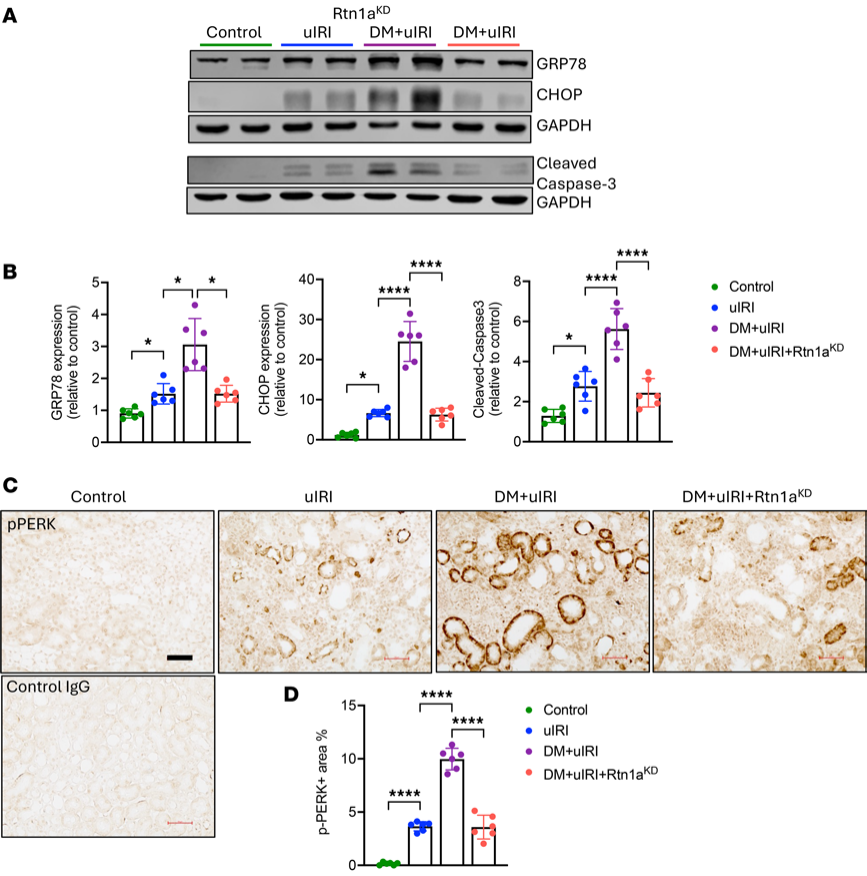
Diabetes predisposes mice to increased ER stress in injured tubules, which is attenuated by RTN1A knockdown. (**A**) Western blot analysis of ER stress (CHOP, GRP78) and apoptosis (cleaved caspase-3) markers in mouse kidney cortices. (**B**) Densitometric analysis of CHOP, GRP78, and cleaved caspase-3 expression (normalized to GAPDH) as a fold change relative to Control. (**C**) Representative immunohistochemistry images of phosphorylated protein kinase R–like ER kinase (p-PERK) in mouse kidneys. Scale bar: 50 μm. (**D**) Quantification of p-PERK^+^ area. *n* = 6 mice, 30 fields evaluated per mouse. *P < 0.05; ****P < 0.0001 between indicated groups by 1-way ANOVA with Tukey’s post hoc test.

We previously demonstrated that RTN1A promotes CKD transition by promoting sustained ER stress and mitochondrial dysfunction by regulating the ER-mitochondria contact (EMC) sites (10, 11, 15). Therefore, we next examined the expression of ER stress and mitochondrial markers in the kidney cortices of mice with uIRI. For ER stress, we examined the expression of ER chaperone protein, glucose-regulated protein 78 (GRP78), also known as BiP, and downstream molecule in the unfolded protein response (UPR), C/EBP homologous protein (CHOP), which triggers apoptosis under sustained UPR and ER stress. We previously showed that GRP78 and CHOP are increased in RTECs in AKI patient kidneys (11). Western blot analysis showed a modest increase in GRP78 expression in kidney cortices of all mice with uIRI compared with healthy controls, but significantly higher levels of GRP78 in DM+uIRI kidneys compared with the other uIRI groups (Figure 5, A and B). Importantly, the expression of CHOP, a marker of the apoptotic response in UPR, and apoptosis marker cleaved caspase-3 was significantly elevated in DM+uIRI kidneys in comparison with uIRI kidneys, indicative of increased RTEC apoptosis in DM+uIRI mice (Figure 5, A and B), while RTN1A knockdown effectively reduced RTEC apoptosis. By immunostaining, we confirmed that these marked changes were occurring in RTECs. As shown in Figure 5, C and D, levels of phosphorylated PKR-like ER kinase (PERK), an ER transmembrane protein kinase that oligomerizes and is autophosphorylated in response to UPR, was not detectable in healthy control kidneys, but increased in the tubules in uIRI kidneys. p-PERK expression was markedly upregulated in the tubules of DM+uIRI mice, but attenuated by RTN1A knockdown, consistent with the above Western blot analysis. These results are also consistent with our previous observation that RTN1A directly interacts with PERK and that its increased expression results in increased PERK and CHOP expression, leading to RTEC apoptosis (10, 11).

**Figure 6:**
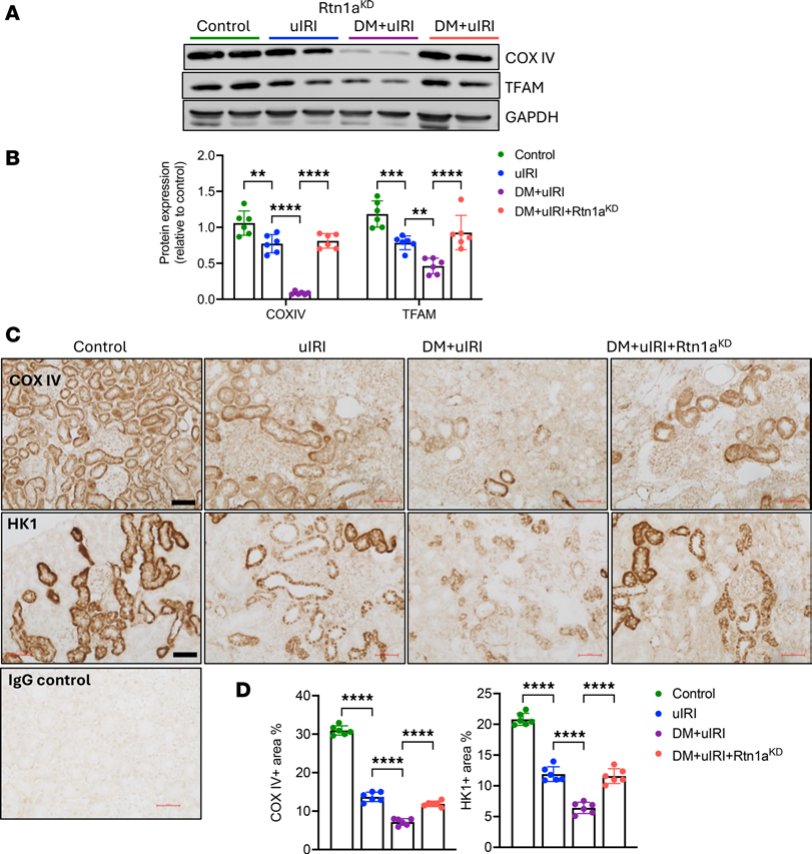
Diabetes predisposes mice to increased mitochondrial damage in injured tubules, which is attenuated by RTN1A knockdown. (**A**) Western blot analysis of mitochondrial markers COX IV and TFAM in the kidney cortices. (**B**) Densitometric analysis of COX IV and TFAM expression (normalized to GAPDH) as a fold change relative to Control. (**C**) Representative immunohistochemistry images of mitochondrial proteins COX IV and HK1 in mouse kidneys. Scale bars: 50 μm. (**D**) Quantification of COX IV^+^ and HK1^+^ areas (%). *n* = 6 mice per group, 30 fields evaluated per mouse. **P < 0.01; ***P < 0.001; ****P < 0.0001 between indicated groups by 1-way ANOVA with Tukey’s post hoc test.

In addition to inducing ER stress, RTN1A was recently identified to mediate ER and mitochondrial crosstalk as a component of EMC sites (15, 24). Increasing evidence supports EMCs as important regulators of mitochondrial homeostasis, apoptosis, and autophagy and that EMC integrity is disrupted in tubular cells in diabetic kidneys (25, 26). We further demonstrated that increased RTN1A expression in RTECs concomitantly worsened ER stress and mitochondrial function in diabetic kidneys (15). Therefore, we next examined the expression of mitochondrial proteins, COX IV and TFAM, by Western blot analysis. As anticipated, a marked reduction in COX IV and TFAM was observed in DM+uIRI kidneys in comparison with the other uIRI groups (Figure 6, A and B). Immunostaining for mitochondrial proteins COX IV and hexokinase-1 (HK1) further corroborated these observations and indicated a marked decrease in mitochondrial proteins by uIRI, which was compounded by underlying diabetes but attenuated by RTN1A knockdown (Figure 6, C and D). Transmission electronic microscopy also revealed markedly altered mitochondrial morphology in kidney cortices of DM+uIRI mice compared with nondiabetic uIRI mice (Supplemental Figure 2), which was markedly reduced by RTN1A knockdown. These results are also consistent with our previous observation that ER-bound RTN1A interacted with mitochondrial HK1, causing its degradation and resulting in mitochondrial dysfunction and the induction of inflammasome and apoptosis pathways in RTECs (15). Thus, RTN1A knockdown restrained the diabetes-mediated aggravation of tubular injury and loss following ischemic injury by reducing ER stress and mitochondrial dysfunction in RTECs.

## Discussion

Although a higher prevalence of AKI in diabetic patients is well recognized, how underlying diabetes may contribute to AKI-to-CKD transition is less well understood. As a growing body of evidence indicates that nonalbuminuric DKD can progress to ESKD, there is a critical unmet need for better elucidation of tubulointerstitial injury in nonalbuminuric DKD. In this study, we sought to first test the hypothesis that unresolved AKI may be the driver of nonalbuminuric DKD transition and that RTN1A is a key mediator of irreversible tubular damage in DKD development, as ER stress and mitochondrial dysfunction are 2 major cellular mechanisms involved in kidney cell injury (27–31), dictating AKI-to-CKD transition (32–34), and in DKD development (16–21). Secondly, although RTN1A’s role in mediating AKI-to-CKD transition in nondiabetic settings and in worsening DKD progression was established independently, whether it is involved in diabetes-induced AKI-to-CKD transition was not known. Therefore, we addressed these 2 questions by using an uIRI model of ischemic injury in diabetic mice with or without RTEC-specific *Rtn1a* shRNA knockdown. We showed that knockdown of *Rtn1a* expression in RTECs ameliorated renal tubular cell injury, fibrosis, and inflammation in diabetic mice with AKI. In addition, *Rtn1a* knockdown also attenuated ER stress, mitochondrial dysfunction, and cell senescence in RTECs in diabetic mice with AKI. These data suggest that RTN1A is a key molecule mediating AKI-to-CKD transition in diabetic mice and that drugs targeting RTN1A could be developed as potential therapies to treat these patients.

Our study has a few limitations: (a) We have not validated our findings in an additional model of diabetes or AKI. We used the HFD-induced obese and STZ-induced diabetic mice as a model of diabetes that encompasses important aspects of both type 1 and type 2 diabetes, as the majority of type 1 diabetic patients develop obesity, and type 2 diabetic patients often have both obesity and insulin deficiency (22, 23). Using this model also allowed us to avoid extensive backcrossing of *Rtn1a*^KD^ mice and crossing with genetically modified diabetic models in DKD-susceptible strains. We used uIRI as an AKI model because ischemia-induced kidney injury is the most common cause of AKI in diabetic patients due to diabetes-induced vasculopathy. However, future studies are required to validate our findings by using different diabetic and AKI models. (b) We could not completely differentiate AKI-induced CKD from diabetes-induced CKD or DKD. We decided to perform uIRI at 4 weeks after induction of diabetes, and all mice were sacrificed at 8 weeks after induction of diabetes to avoid a substantial phenotype change from DKD. Indeed, these diabetic mice did not have much glomerular hypertrophy and mesangial expansion, suggesting that they had not fully developed DKD. Therefore, CKD is likely caused by AKI, and mild elevation of albuminuria is likely caused by tubular injury. Future studies are required to determine how AKI induces CKD in mice with a DKD phenotype. (c) We used *Rtn1a*^KD^ mice instead of *Rtn1a*-knockout mice. While knockout mice may give us clearer phenotypes, knockdown mice might be more useful for therapeutic purposes because most drugs do not block target molecules completely. (d) RTN1A may act as a convergence point for various pathways involved in both DKD and the AKI-to-CKD transition. For instance, recent evidence suggests that glycogen synthase kinase 3 (GSK3), particularly the β isoform, is an important mediator in the pathogenesis of both DKD (35–37) and AKI-to-CKD transition (38, 39). Interestingly, a recent study of interrogating the interactomes of GSK3 using affinity purification and proximity-dependent biotinylation mass spectrometry indicates that GSK3 interacts with RTN1 (40), suggesting that RTN1 may mediate the pathogenic role of GSK3 in both DKD and in AKI-to-CKD transition. (e) Although the study suggests that diabetes-induced AKI-to-CKD transition could contribute to nonalbuminuric DKD, direct evidence is still lacking, and other factors or mechanisms may also contribute to the development of nonalbuminuric DKD. (f) Other factors than RTN1A may also contribute to the AKI-to-CKD transition in the context of diabetes, and future studies are required to identify these pathological factors.

In summary, our key findings indicate that diabetes can aggravate AKI-to-CKD transition, leading to accelerated renal tubulointerstitial injury and fibrosis, and that ER stress, mitochondrial dysfunction, and senescence of RTECs are key events mediating diabetes-induced aggravation of AKI-to-CKD transition. Our study also highlights the pivotal role of RTN1A in linking ER stress and mitochondrial dysfunction in RTECs, as its knockdown in RTECs ameliorates AKI-to-CKD transition in diabetic mice. Our study suggests that AKI-to-CKD transition may contribute to the development and progression of nonalbuminuric DKD in diabetic patients, and RTN1A inhibitors could potentially be developed as a potential therapy to treat these patients.

## Methods

### Sex as a biological variable.

We used male mice for the study, as they exhibit less variability in DKD phenotypes.

### Mouse model.

The tetracycline-inducible RTEC-specific *Rtn1a*^KD^ mouse model (Pax8-rtTA;*Rtn1a*^KD^) was previously described (10, 11, 14). To induce *Rtn1a* knockdown, mice were given doxycycline-supplemented drinking water (500 ng/mL) for the duration, as indicated. Diabetes was induced in 8-week-old male double transgenic Pax8-rtTA;*Rtn1a*^KD^ (mixed C57BL/6J strain) or age- and sex-matched control littermate mice with a single transgene by administration of 60% HFD (Bio-Serv) for 12 weeks, followed by low-dose STZ (50 mg/kg) injection for 5 consecutive days. Nondiabetic mice were given a normal diet. AKI was induced by uIRI by clamping of the right renal pedicle for 30 minutes at 37°C. To assess kidney function in mice subjected to uIRI, the contralateral kidney was removed 1 day before euthanasia. All mice were euthanized after 20 weeks of each diet for collection of blood, urine, and kidney samples.

### Urine albumin and creatine measurements.

Urinary albumin excretion was assessed from collected urine samples at indicated times (Figure 2A) and expressed as the albumin-to-creatine ratio (mg/mg). Urine albumin was quantified by a commercially available ELISA kit (Bethyl Laboratories, E99-134), and creatinine levels were measured in the same samples using a creatinine assay kit (BioAssay Systems, DICT-500).

### Blood glucose and BUN measurements.

Blood glucose was measured using a blood sample from the tail vein using a glucometer at indicated times (Accu-Chek Aviva). Because the glucometer’s maximum upper limit is 600 mg/dL, in cases when the maximal values were obtained, the actual glucose level may have been higher in mice. BUN was measured from mouse sera at the endpoint using a commercially available kit (BioAssay Systems, DIUR-100).

### Kidney histology and tubular injury scoring.

Mouse kidneys were fixed using freshly prepared 4% formaldehyde solution and processed for cryopreservation or paraffin embedding. Paraffin-embedded sections (4 μm) were stained with PAS for histologic analysis. The mesangial and glomerular cross-sectional areas were assessed on a minimum of 40 glomeruli per mouse by the operator blinded to the experimental groups on ×200 and ×400 magnification images (Zeiss AX10 microscope, Carl Zeiss). The tubular injury was scored in the cortical regions on a scale from 0–4, based on tubular dilatation, loss of brush border and tubular structure, and tubular atrophy: 0 (normal); 1 (less than 25%); 2 (25%–50%); 3 (50%–75%); and 4 (more than 75%). Scoring was performed on a minimum of 30 cortical fields (×200 original magnification) per PAS-stained kidney section per mouse and averaged (*n* = 6 mice per group). The nonparametric Kruskal-Wallis test with Dunn’s post hoc test was applied for statistical analysis of tubular injury scores. Paraffin-embedded kidney sections were also stained with Masson’s trichrome and picrosirius red with fast green to evaluate kidney fibrosis according to the manufacturer’s instructions (Abcam, ab150681 and Thermo Fisher Scientific, 87019). The staining intensities were quantified by ImageJ (NIH).

### Transmission electron microscopy.

Kidney tissues were fixed in 2.5% glutaraldehyde with 0.1 M sodium cacodylate (pH 7.4) for 72 hours at 4°C. Samples were further incubated with 2% osmium tetroxide and 0.1 M sodium cacodylate (pH 7.4) for 1 hour at room temperature. Ultrathin sections were stained with lead citrate and uranyl acetate and viewed on a Hitachi H7650 microscope. The morphology of mitochondria was examined in the kidneys of the mice. Quantification of dysmorphic mitochondria was performed as previously described (15, 41). Briefly, an average of 50 mitochondria were analyzed per mouse kidney (*n* = 3 mice per group), and dysmorphic mitochondria were defined as mitochondria with a focal loss of visible cristae, clustering of residual cristae at the peripheral mitochondrial membrane, and fragmented (<2 μm in length).

### Western blot analysis.

Mouse kidney tissues were processed in a lysis buffer containing 1% NP-40 with a protease and phosphatase inhibitor cocktail. Following primary antibodies were used: anti–α-SMA (Abcam, ab7817), anti-MMP2 (Abcam, ab97779), anti-fibronectin (Cell Signaling Technology, 26836s), anti-GRP78 (Santa Cruz Biotechnology, sc-166490), anti-CHOP (Cell Signaling Technology, 2895), anti-TFAM (Abcam, ab131607), anti–COX IV (Cell Signaling Technology, 4850), anti-HK1 (Cell Signaling Technology, 2024), anti–cleaved caspase-3 (Cell Signaling Technology, 9664), anti-vimentin (Cell Signaling Technology, 5741), anti–E-cadherin (BD Biosciences, 610182), and anti-GAPDH (Cell Signaling Technology, 2118). Western blots were developed with an enhanced chemiluminescence system and imaged with Odyssey Fc Imaging System (LI-COR Biosciences). For densitometric analysis, the intensity of each target protein was normalized to GAPDH. All experiments were repeated at least 3 times, and representative experiments are shown.

### Immunostaining.

Immunofluorescent staining was performed on frozen mouse kidney tissues using anti-RTN1A (MON162, Abcam, ab9274), anti-F4/80 (Invitrogen, 14480182), anti-p21 (Abcam, ab188224), and anti–γ-H2A.X (Abcam, ab81299). Immunohistochemistry was performed on formalin-fixed, paraffin-embedded kidney sections. Sections were deparaffinized and heated in Tris-EDTA buffer (pH 9) for antigen retrieval. Sections were incubated with hydrogen peroxide to inhibit endogenous peroxidase activity, blocked with diluted donkey serum for 1 hour at room temperature, and incubated with primary antibodies overnight at 4°C. Primary antibodies include anti–p-PERK (Santa Cruz Biotechnology, sc-32577), anti–COX IV (Cell Signaling Technology, 4850), and anti-HK1 (Cell Signaling Technology, 2024). The sections were washed and processed using Vectastain Elite ABC kit (Vector Laboratories, PK-6100). Images were acquired using an AxioVision II microscope with a digital camera (Carl Zeiss). For quantification of immunostaining, 30 nonoverlapping fields were randomly selected per mouse kidneys and quantified using ImageJ software.

### Quantitative real-time PCR.

Primers for RT-PCR were designed using the Primer-Blast (NCBI) tool. PCR was performed using SYBR Green Master Mix (Applied Biosystems) and the Applied Biosystems 7500 Real-Time PCR system. Gene expression was normalized to the housekeeping gene GAPDH, and fold changes in expression relative to the control group were calculated using the 2^–ΔΔCt^ method.

### Statistics.

Data are expressed as mean ± SD. The distribution of data was assessed with the Shapiro-Wilk and Kolmogorov-Smirnov normality tests. One-way or 2-way ANOVA followed by Tukey’s post hoc test or Kruskal-Wallis nonparametric test with Dunn’s post hoc test was used for comparisons between groups, as appropriate. A *P* value of less than 0.05 was considered statistically significant. GraphPad Prism software (v.10) was used for all statistical analysis.

### Study approval.

All mouse studies were performed under the guidelines approved by the Institutional Animal Care and Use Committee at the Icahn School of Medicine at Mount Sinai (IACUC-LA10-00001).

### Data availability.

All data are available from the corresponding author upon request. Raw data for all graphs can be found in the Supporting Data Values file.

## Author contributions

JCH and KL designed the research project. LM, YC, and FZ performed the experiments. JCH, KL, LM, and YC analyzed the data. LG and ZN participated in the discussion of the data. JCH, KL, and LM drafted and revised the manuscript. The number of experiments performed by each researcher was used to determine the order of the 2 co–first authors. All authors approved the final version of the manuscript.

## Supplementary Material

Supplemental data

Unedited blot and gel images

Supporting data values

## Version 1 12/20/2024

Electronic publication
