# Supplementary material for: RTN1A mediates diabetes-induced AKI-to-CKD transition
Source: JCI Insight. 2024 Dec 20;9(24):e185826. doi: 10.1172/jci.insight.185826 (PMC11665580; doi:10.1172/jci.insight.185826)
Supplement: Supplemental data [file jciinsight-9-185826-s204.pdf]

Supp. Figure 1

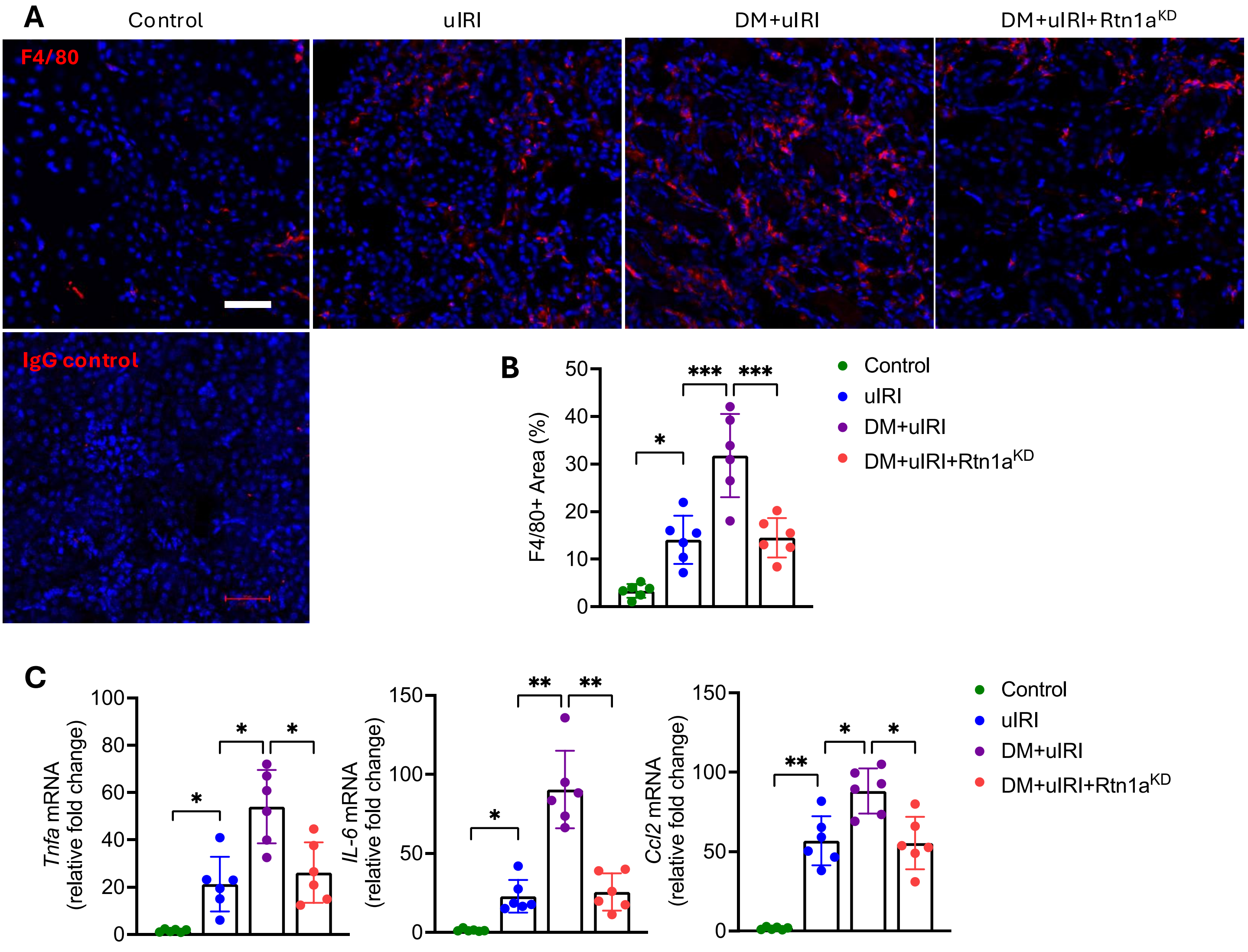

Supp. Fig. 1: Diabetes exacerbates renal inflammation in mice with uIRI, which is attenuated by RTN1A knockdown. A. Representative images of F4/80 immunostaining, Scale bar, 50μm. **B.** Quantification of F4/80 area per mouse kidney (%). 30 fields evaluated per mouse. **C**. Real-time PCR analysis of inflammatory markers in mouse kidney cortices. n=6 mice, *P<0.05, **P<0.01, and ***P<0.001 between indicated groups by 1-way ANOVA with Tukey's *post hoc* test.

Supp. Figure 2

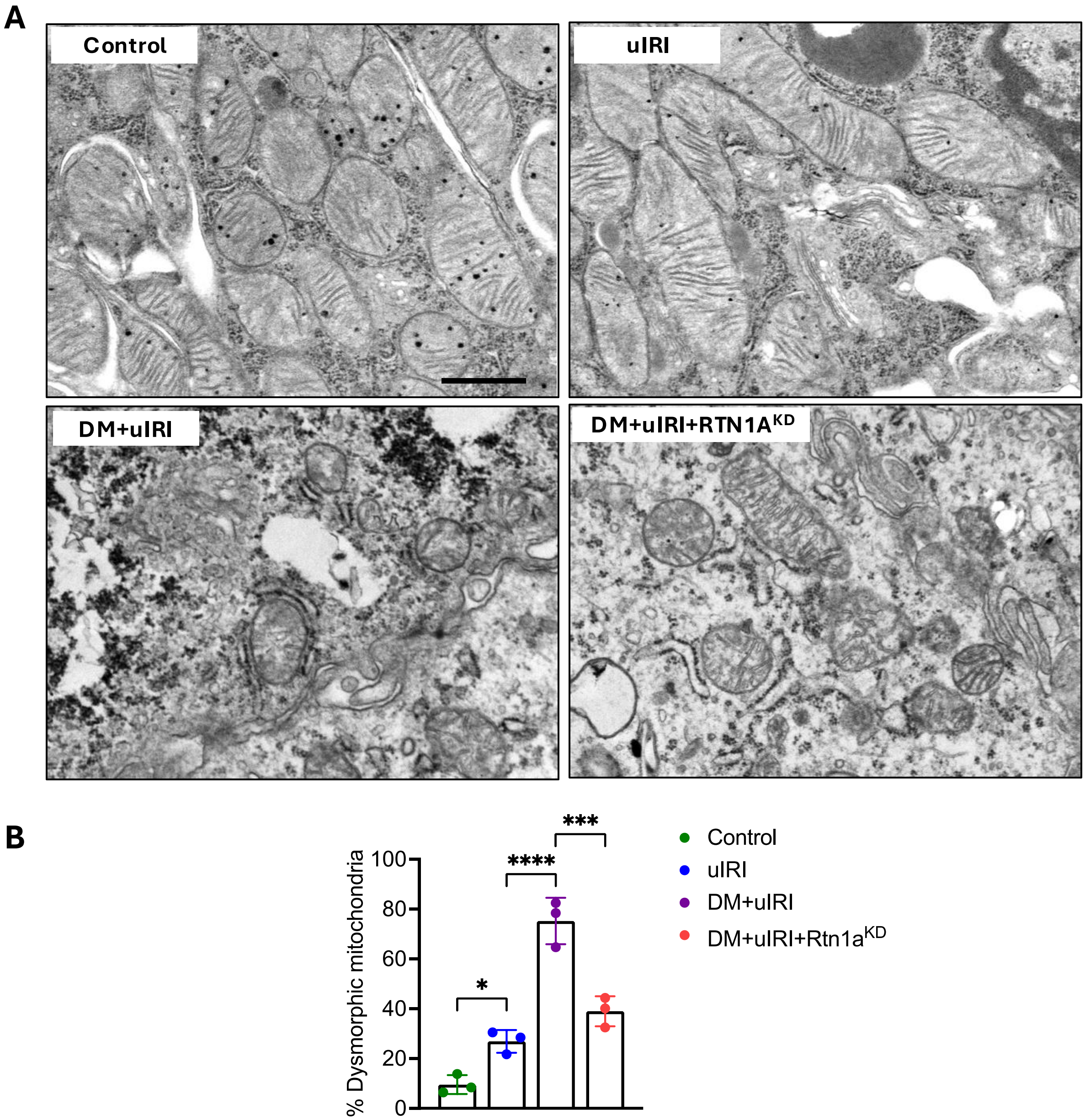

Supp. Fig. 2: Changes in mitochondria morphologies in diabetic mice with uIRI are attenuated by RTN1A knockdown. A. Representative transmission EM images of RTEC mitochondria in 4 groups of mice, Scale bar, 600nm. **B.** Average % of dysmorphic mitochondria in RTECs per mouse kidney. n=3 mice, **P*<0.05, ****P*<0.001, and *****P*<0.0001 between indicated groups by 1-way ANOVA with Tukey's *post hoc* test.
