# Supplementary figures and images for: RTN1A mediates diabetes-induced AKI-to-CKD transition
Source: JCI Insight. 2024 Dec 20;9(24):e185826. doi: 10.1172/jci.insight.185826 (PMC11665580; doi:10.1172/jci.insight.185826)

Supplemental file – uncropped WB images

3D:

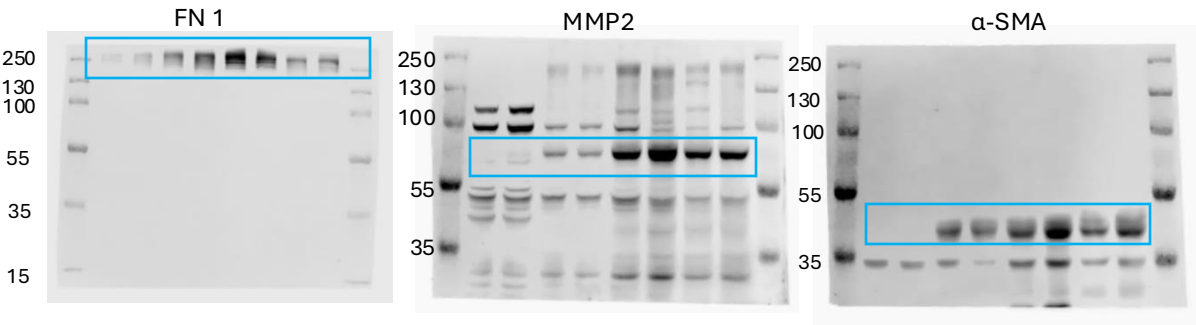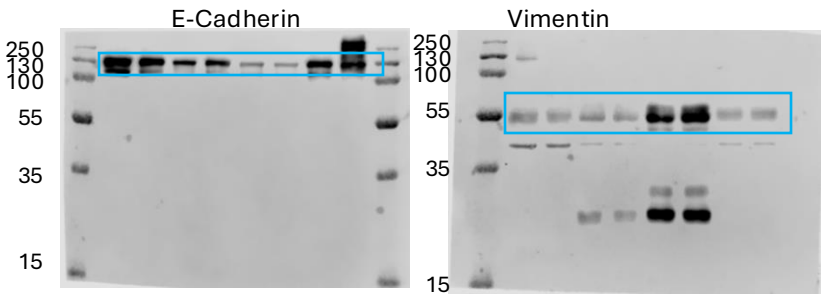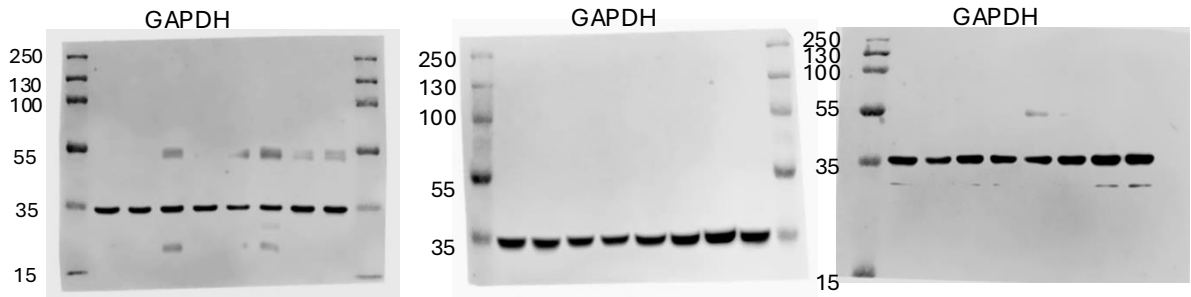

5A:

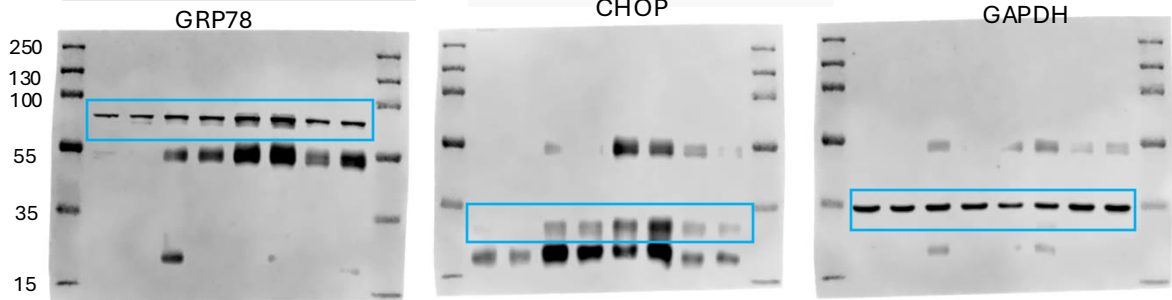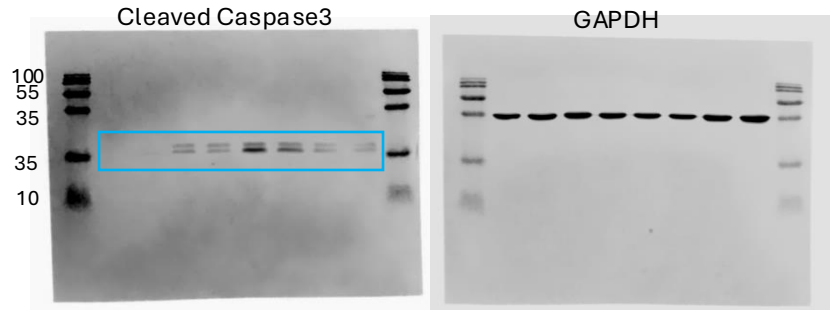

6A:

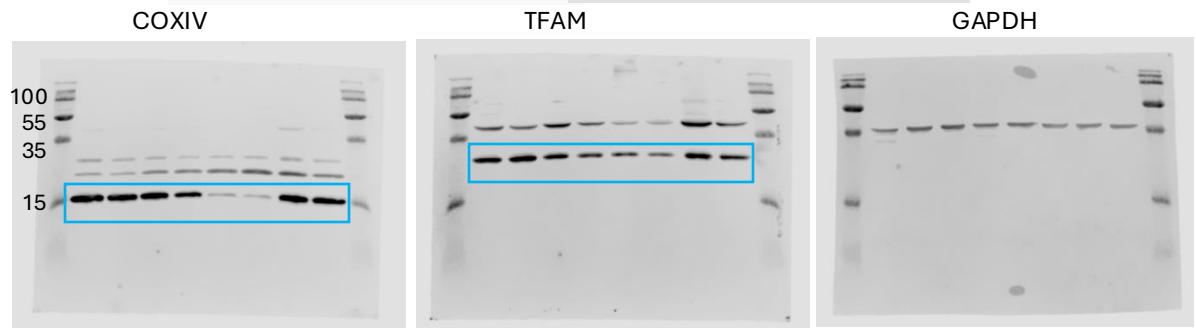

Supplement: Unedited blot and gel images [file jciinsight-9-185826-s205.pdf]
